# The relationships between water storage and biomass components in two conifer species

**DOI:** 10.7717/peerj.7901

**Published:** 2019-10-14

**Authors:** Lai Zhou, Sajjad Saeed, Yujun Sun, Bo Zhang, Mi Luo, Zhaohui Li, Muhammad Amir

**Affiliations:** State Forestry Administration Key Laboratory of Forest Resources & Environmental Management, Beijing Forestry University, Beijing, China

**Keywords:** Water content ratio, Dry mass, Regression analyse, Chinese fir, Korean larch

## Abstract

**Background:**

Water storage is a significant physiological index of vegetation growth. However, information on water storage at the individual tree level and its relationship to climatic conditions and productivity is scarce.

**Methods:**

We performed a comparative analysis of water storage using field measurements acquired three age classes of Chinese fir (*Cunninghamia lanceolata*) and Korean larch (*Larix olgensis*). The distributions of water storage, water content ratio and dry mass were presented, and regression analyses were used to confirm the relationships of water storage and water content ratio to dry mass components, respectively.

**Results:**

Our results indicated that water was mostly concentrated in the stem xylem, which aligned well with the distribution of dry mass in both conifer species. However, the water storage of the stem xylem was always higher in Chinese fir than in Korean larch. The average water content ratio of both conifer species decreased with age, but that of Chinese fir was always higher than that of Korean larch. There was a significant difference in the water storage proportion in the components of Chinese fir (*P* < 0.001) and Korean larch (*P* < 0.001). The effects of age class on the water storage of Chinese fir (*P* = 0.72) and Korean larch (*P* = 0.077) were not significant. Interestingly, significant positive linear correlations were found between fine root water and leaf water and mass in Chinese fir (*P* < 0.001, *R*^2^ ≥ 0.57) and Korean larch (*P* < 0.001, *R*^2^ ≥ 0.74). The slopes showing that the linear relationship between tree size and water content ratio of stem xylem were always steeper than that of other components for the two conifers.

**Conclusion:**

Our study indicates the similar water related characteristics and their close relations to biomass accumulation and growth in both fast growing species at contrasting climates, illustrating the same coherent strategies of fast growing conifers in water utilization.

## Introduction

Plant water storage plays a crucial role in physiological processes and biomass accumulation (Lisar et al., 2012; Hoeber et al., 2014). Plants in different environments have different biophysiological responses and water use strategies to balance the needs of survival and growth (Von Allmen, Sperry & Bush, 2015; Meinzer, 2016; Bongers et al., 2017; Jia et al., 2017; Zhu et al., 2017; Conte et al., 2018). In arid environments, plants lower their relative water storage, physiological and biochemical processes, and sacrifice growth for survival (Bota, Medrano & Flexas, 2004; Flexas, Escalona & Medrano, 2010; Medrano et al., 2015; Mendiguren et al., 2015; Moshelion et al., 2015; Šímová, Rueda & Hawkins, 2017). In order to survive, plants modify their morphological features to reduce water loss (Paz, Pineda-García & Pinzón-Pérez, 2015; Venegas-González et al., 2015; Longuetaud et al., 2016). Different plant species also have different water use strategies when adapting to environmental conditions (David et al., 2007; Hernández et al., 2010; Liu et al., 2018; Tang et al., 2018; Werden et al., 2018).

The space-time change in soil water storage is one environmental factor that has been thoroughly studied (Hu et al., 2008; Xu et al., 2017a, 2017b; Zhao et al., 2017). Although the water storage of vegetation also plays an important ecohydrological role (Fan et al., 2012; Hoeber et al., 2014), it has not received sufficient attention, partly because its study involves destructive methods. Plant water storage and its dynamics are different based on soil water availability, tree species, tree size, wood properties, drought tolerance, and hydraulic strategy (Oliva Carrasco et al., 2014; Longuetaud et al., 2016; Matheny et al., 2016). Most other studies have focused on water storage quantities, measurement method, component connection, and species-specific water storage characteristics (Clevers, Kooistra & Schaepman, 2010; Longuetaud et al., 2017; Matheny et al., 2016; Paz-Kagan & Asner, 2017; Saito et al., 2016). Researchers have reported that trunk storage appears to provide a buffer to water demands during transpiration (Matheny et al., 2016). A recent study also indicated that trunk water storage was strongly coordinated with ecophysiological traits such as growth rate (Oliva Carrasco et al., 2014). However, the volume of stored water was shown to vary with tree size and species-specific hydraulic properties (wood density, drought tolerance, and stomatal hydraulic strategy) (Matheny et al., 2016). A better understanding of the relationship between water storage and biomass components, which would increase our ability to predict how water storage interacts with biomass productivity, is required, especially for fast growing conifers. To our best knowledge, no study has been conducted to outline the relationships between water storage and biomass components and to explore the coherent water utilization strategies in Chinese fir (*Cunninghamia lanceolata*) and Korean larch (*Larix olgensis*).

There were many studies on canopy water storage (Clevers, Kooistra & Schaepman, 2010; Paz-Kagan & Asner, 2017) and stem water storage (Wullschleger, Hanson & Todd, 1996; Saito et al., 2016; Longuetaud et al., 2017). However, reported data were often obtained remotely (Saatchi & Moghaddam, 2000; Zhou et al., 2018b), and there has been a lack of field research in component water traits and their interaction with biomass accumulation, growth, and productivity. Additionally, most of the previous studies focused on different tree species in the same climatic environment (Hoeber et al., 2014; Longuetaud et al., 2016, 2017). Different species in different ecosystems might have similar hydraulic mechanisms (Domec et al., 2010; Yu & D’Odorico, 2015). Because of the strong relationship between water storage and plant physiological processes (Guada et al., 2018), we hypothesized that the two conifers growing in contrasting climates would have similar water storage distribution characteristics, due to their similar growth habits, and such characteristics are closely related to growth and therefore biomass accumulation. Our study focused on Chinese fir and Korean larch, which are two conifers adapted to warm and moist, and cold and dry climates in China, respectively. We performed comparative analyses of water content characteristics of these two conifers and explored their relationships to biomass components at tree level with stand development.

## Materials and Methods

### Site description

**Figure 1:**
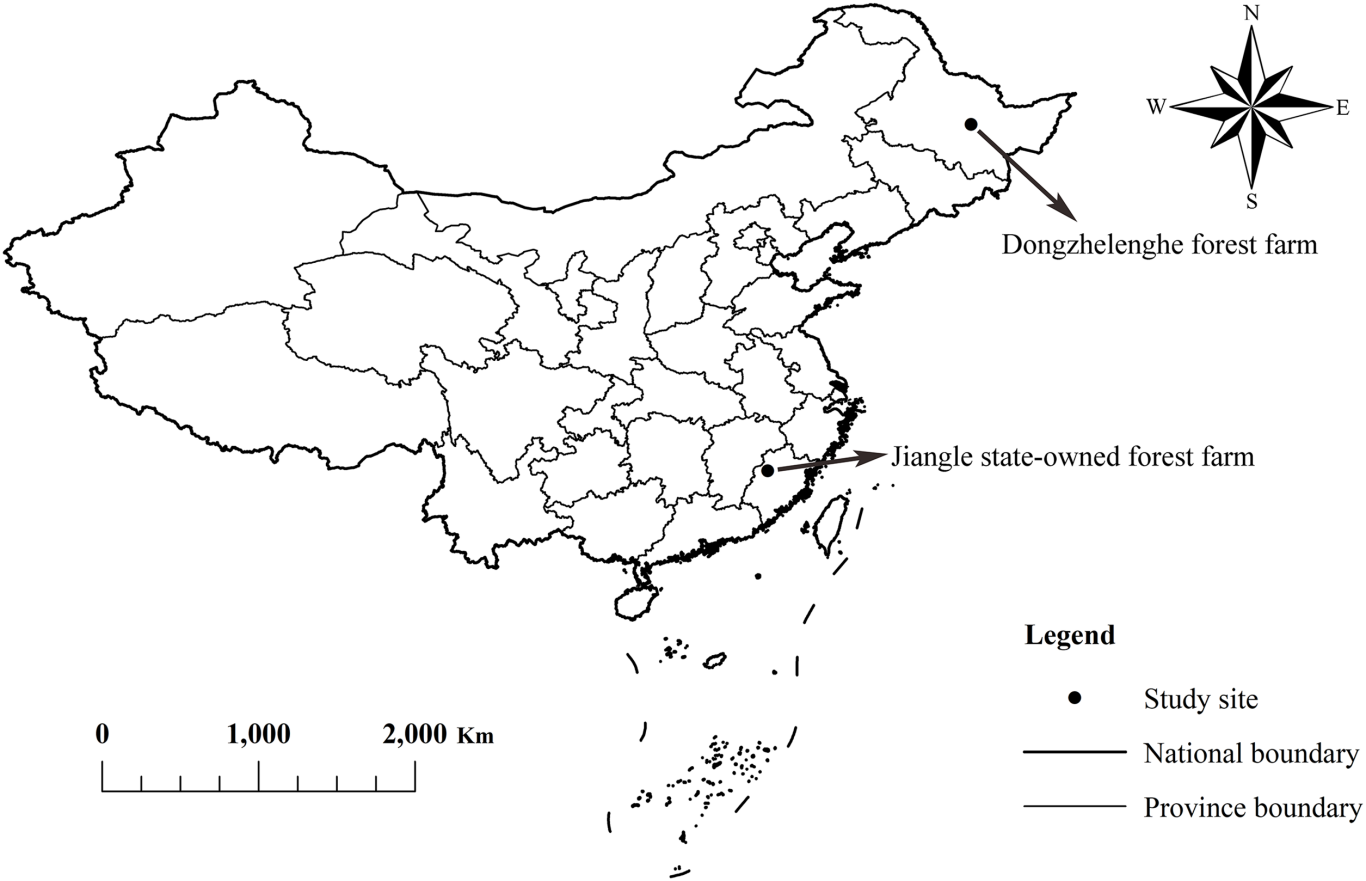
Study sites in Southeast and Northeast China. Each black point indicates the location of the study sites. Dongzhelenghe forest farm is the study site for 12 Korean larch, and Jiangle state-owned forest farm is for 12 Chinese fir. The black lines indicate the national or province boundary. Dongzhelenghe forest farm is in Heilongjiang Province. Jiangle state-owned forest farm is in the Fujian Province.

The study was carried out at the Jiangle state-owned forest farm (JF) in Fujian Province, Southeast China, and the Dongzhelenghe forest farm in Heilongjiang Province (DF, latitude 46°31′−46°49′N, longitude 128°55′−129°15′E), Northeast China (Fig. 1). Jiangle County (latitude 26°26′−27°04′N, longitude 117°05′−117°40′E) is representative of Southern China forest regions, and its forest coverage is 85.2%. The elevation of the study site is approximately 200–500 m above mean sea level. The region is situated in a typical humid tropical monsoon climate, with an average annual precipitation of 1,910 mm and an annual mean temperature of 18.2 °C. Frost rarely occurs in this area. The topography of the region is low mountains and hilly landforms. The soil is clay loamy and red, developed from shale and slate parent rocks. The dominant tree species is Chinese fir, but Masson pine (*Pinus massoniana*), *Schima superba* (*Schima superba*), and bamboo species (*Bambusoideae*) are also found (Guangyi, Yujun & Saeed, 2017; Liping, Yujun & Saeed, 2018; Saeed et al., 2019). DF is located in the southern foothills of the Xiaoxinganling Mountains, Yichun city, in the Northeast China forest region. The forest farm is mainly composed of fast-growing, high-yield forests of Korean larch. The topography is low hills with an elevation of 260–410 m. The area is situated in a typical northern temperate continental climate, with an annual mean temperature of 1.0 °C, ranging from an average of −22.4 °C during the coolest month (January) to 21.0 °C during the warmest month (July). The mean annual precipitation is 750–820 mm, occurring mostly between June and August. The frost-free period is 110–125 days. The soil is dark brown, a typical forest soil in Northeast China, with a thickness of 30–60 cm and a little gravel (Ma et al., 2014; Fu et al., 2016).

### Sampling and processing

A total of 24 trees (representing young, medium, and old ages) for both sites were destructively sampled from 12 permanent sample plots of the Chinese fir plantation in JF during July 2017, and 12 permanent sample plots of the Korean larch plantation in DF during July 2018. The plots were different ages. The 2 years were normal for the two sample sites. Meteorological data from the National Meteorological Information Centre (http://data.cma.cn/) showed that the July temperature and precipitation were 30.2 °C and 296.3 mm, respectively, at JF in 2017, and 23.5 °C and 161.9 mm at DF in 2018. The plot size was 20 × 30 m. The stand characteristics of the two conifers are presented in Table 1. Tree age was checked against diameter at breast height (DBH, 1.3 m) and the management records of the forest farms. According to the historical records, the stands at the two study sites were both second-generation plantations.

**Table 1:** Stand characteristics of the two study species.

Species	Age class	Average DBH (cm)	Average H (m)	Stand density (n · hm^−2^)	Basal area (m^2^ · hm^−2^)
Chinese fir	Young	6.6	7.0	2,800.2	4.8
	Medium	16.6	15.8	1,503.1	23.2
	Old	24.7	22.4	933.3	36.4
Korean larch	Young	5.3	5.4	2,011.8	5.2
	Medium	15.0	15.3	1,255.7	20.1
	Old	22.9	19.7	780.8	31.5

Sampled trees were measured for diameter and total height after felling with a mechanical chain saw. Stems, live branches, dead branches, and roots were separated and recorded for fresh weights immediately in the field using an electronic scale (to either nearest 0.1 kg or 10 mg). All live and dead branches were measured for basal diameter, branch length, extent width, branch angle, and depth into crown. Stems were cut into one m sections and two to three branches (live and dead) of average size in each section were bagged in plastic bags for determination of water storage and biomass. The tree top (top section < 1 m) was treated as a branch. Stem discs of four cm in thickness were taken at the small end of each section and determined for fresh weight with and without bark. Bark samples of approximately 50 g for each disc were randomly taken and placed into a paper bag for determination of water storage and biomass. In the lab, leaves were removed from sample branches and recorded for fresh weight. About 50 g samples were taken separately for leaves and bare branch for determination of water storage and biomass of each sample branch.

Whole roots were carefully excavated with shovels within the projected area of tree crown and brought back to the lab to determine fresh and oven-dried weight (Wang et al., 2015). Stump samples were taken by saw (approximately 50 g each stump) and roots were sampled by soil depth of 0–20, 20–40, 40–60, and 60–80 cm. Within each soil layer, roots were separated into large (>20 mm), coarse (5–20 mm), medium (2–5 mm), and fine (<2 mm) classes. The roots of each root class in each soil layer were recorded for fresh weight and sampled (approximately 50 g each sample) for fresh weight and dry mass.

The fresh samples of each component were oven-dried at 105 °C for 48 h to a constant weight before dry mass was recorded with an electronic scale (Longuetaud et al., 2017).

### Calculation of water storage, water content ratio and dry mass

The water content ratio of each sample was calculated as a ratio of water weight (difference between fresh weight and dry mass) to fresh weight as follows:

(1) $${\rm WCR_{sample} = 1}{\rm - }\displaystyle{{{\rm DM_{sample}}} \over {{\rm FW_{sample}}}}$$

Where WCR_sample_ is sample water content ratio, DM_sample_ is sample dry mass, and FW_sample_ is sample fresh weight.

The water storage and dry mass of sample stem were calculated using Eqs. (2) and (3).

(2) $${\rm{W}}{{\rm{S}}_{{\rm{leaf }}\,{\rm{or}}\,\,{\rm{ bare}}\,\,{\rm{ branch}}}} = \sum\limits_{j = 1}^m {\bigg(\!\!\bigg(\sum\limits_{i = 1}^n {{\rm{F}}{{\rm{W}}_{{\rm{leaf }}\,{\rm{or}}\,\,{\rm{ bare}}\,\,{\rm{branch}}}}/n\bigg) \times N \times {\rm{WC}}{{\rm{R}}_{{\rm{leaf}}\,{\rm{ or}}\,\,{\rm{bare}}\,\,{\rm{branch}}}}\bigg)} } $$

(3) $${\rm{D}}{{\rm{M}}_{{\rm{leaf}}\,{\rm{or}}\,\,{\rm{bare}}\,\,{\rm{branch}}}} = \sum\limits_{j = 1}^m {\bigg(\!\!\bigg(\sum\limits_{i = 1}^n {{\rm{F}}{{\rm{W}}_{{\rm{leaf}}\,{\rm{or}}\,\,{\rm{bare}}\,\,{\rm{branch}}}}/n\bigg)\!\times N\!\times \bigg(1 - {\rm{WC}}{{\rm{R}}_{{\rm{leaf}}\,{\rm{or}}\,\,{\rm{bare}}\,\,{\rm{branch}}}}} }\bigg)\!\!\bigg)$$

where WS_leaf or bare branch_ is leaf (or bare branch) water storage of sample stem, FW_leaf or bare branch_ is leaf (or bare branch) fresh weight of the *n*th sample branch, *n* is the number of sample branches within each stem section, *N* is the number of total branches within each stem section, WCR_leaf or bare branch_ is the water content ratio of the leaf (or bare branch) samples in each stem section, *m* is the number of stem section of each sample tree, and DM_leaf or bare branch_ is leaf (or bare branch) dry mass of sample tree.

Water storage and dry mass were calculated separately for roots and stumps with Eqs. (4) and (5), and for fine roots with Eqs. (6) and (7):

(4) $${\rm{W}}{{\rm{S}}_{{\rm{root}}}}{\rm{ = }}\sum\limits_{j{\rm{ = 1}}}^{\rm{4}} {\sum\limits_{i{\rm{ = 1}}}^{\rm{4}} {{\rm{(F}}{{\rm{W}}_{{\rm{(di,}}\,{\rm{layer}}\,j{\rm{)}}}}} \, \times } \,{\rm{W}}{{\rm{S}}_{{\rm{(di,}}\,{\rm{layer}}\,j{\rm{)}}}}\, + \,{\rm{F}}{{\rm{W}}_{{\rm{stump}}}}\,{\rm{ \times }}\,{\rm{WC}}{{\rm{R}}_{{\rm{stump}}})}$$

(5) $${\rm{D}}{{\rm{M}}_{{\rm{root}}}}{\rm{ = }}\sum\limits_{j{\rm{ = 1}}}^{\rm{4}} {\sum\limits_{i{\rm{ = 1}}}^{\rm{4}} {{\rm{(F}}{{\rm{W}}_{{\rm{(di,}}\,{\rm{layer}}\,j{\rm{)}}}}\,{\rm{ \times }}\,\,{\rm{(1}} - {\rm{WC}}{{\rm{R}}_{{\rm{(di,}}\,{\rm{layer}}\,j{\rm{)}}}}{\rm{)}}} {\rm{)}}} \,{\rm{ + }}\,{\rm{F}}{{\rm{W}}_{{\rm{stump}}}}\,{\rm{ \times }}\,\,{\rm{(1}} - {\rm{WC}}{{\rm{R}}_{{\rm{stump}}}}{\rm{)}}$$

where WS_root_ is the root water storage of the sample tree, FW_(di, Layer__*j*)_ is the root fresh weight by diameter class (*d1* = 0–2 mm, *d2* = 2–5 mm, *d3* = 5–20 mm, *d4* > 20 mm) and soil layer (*j1* = 0–20 cm, *j2* = 20–40 cm, *j3* = 40–60 cm, *j4* = 60–80 cm), WCR_(di, Layer__*j*)_ is the root water content ratio by diameter class and soil layer, FW_stump_ is the stump fresh weight, WCR_stump_ is the stump water content ratio, and DM_root_ is the root dry mass of sample tree.

(6) $${\rm{W}}{{\rm{S}}_{{\rm{fine}}\,{\rm{root}}}} = \sum\limits_{j{\rm{ = 1}}}^{\rm{4}} {{\rm{(F}}{{\rm{W}}_{{\rm{(di,}}\,{\rm{layer}}\,j{\rm{)}}}}} {\rm{ \times WC}}{{\rm{R}}_{{\rm{(di,}}\,{\rm{layer}}\,j{\rm{)}}}})\,({\rm di} = 0\!-\!2\,{\rm{mm}})$$

(7) $${\rm{D}}{{\rm{M}}_{{\rm{fine}}\,{\rm{root}}}}{\rm{ = }}\sum\limits_{j{\rm{ = 1}}}^{\rm{4}} {{\rm{(F}}{{\rm{W}}_{{\rm{(di,}}\,{\rm{layer}}\,j{\rm{)}}}}} \, \times \,({\rm{1}} - {\rm{WC}}{{\rm{R}}_{{\rm{(di,}}\,{\rm{layer}}\,j{\rm{)}}}}{\rm{)}})\,({\rm{di}} = \,0\!-\!2{\rm{mm}})$$

where WS_fine root_ is the fine root water storage and DM_fine root_ is the fine root dry mass of the sample tree.

The average stem xylem and bark water content ratios of the sample tree were calculated from those of various stem sections.

### Statistical analysis

The data were log-transformed to ensure that the residuals were normally distributed. Statistical comparisons of water storage, dry mass proportion, and water content ratio were conducted using SPSS 20.0 software (a two-way ANOVA test: age class and biomass component). Tukey’s multiple comparison procedure was used to assess differences between age classes or biomass components. The differences were considered statistically significant when *P* < 0.05. Linear regression analyses were used to analyze the relationship between water storage (or water content ratio) and dry mass using Origin Pro 2016. Origin Pro 2016 was also used to display the statistical results.

## Results

### Water storage distribution

The water storage proportion differed among different biomass components (*P* < 0.001 for both Chinese fir and Korean larch), which varied with age class in Korean larch, especially for leaves and stem xylem (significant biomass component and age class interaction, *P* < 0.001). The water storage proportion was the highest in stem xylem (30–70%) and varied between 10% and 20% for leaves, live branches, stem bark, and roots in both conifers, but was the lowest in dead branches (<2%) (Table 2). The stem xylem water proportion of Chinese fir increased with age class, while that of Korean larch reached the maximum at medium age, although the differences among age classes were not statistically significant in both species. The variation of water storage proportions with age class was similar between leaves and live branches, generally decreasing with age in both conifers. Among the biomass components, the root and stem bark water storage proportions showed the smallest differences among age classes and between species.

**Table 2:** Water fraction (%) of Chinese fir and Korean larch at different age classes.

Species	Age class	Leaf	Live branch	Dead branch	Stem bark	Stem xylem	Root system
Chinese fir	Young	16.11 ± 2.75^b^	9.88 ± 2.70^c^	0.00 ± 0.00^d^	6.97 ± 2.94^c^	53.57 ± 6.13^a^	13.47 ± 3.44^bc^
	Medium	7.97 ± 1.47^b^	6.55 ± 1.22^b^	0.39 ± 0.33^c^	8.38 ± 0.72^b^	60.88 ± 7.67^a^	15.83 ± 4.61^b^
	Old	2.66 ± 0.75^cd^	5.40 ± 1.08^b^c	1.13 ± 1.43^d^	8.64 ± 0.24^b^	69.08 ± 4.19^a^	13.08 ± 3.76^b^
Korean larch	Young	19.78 ± 2.60^b^	16.53 ± 0.70^b^	0.13 ± 0.11^d^	9.25 ± 1.21^c^	34.04 ± 4.26^a^	20.27 ± 2.83^b^
	Medium	4.44 ± 1.02^c^	9.41 ± 2.63^b^	1.26 ± 1.04^d^	9.24 ± 1.40^b^	60.38 ± 3.87^a^	15.28 ± 4.74^b^
	Old	7.89 ± 1.54^cd^	11.88 ± 1.03^c^	0.37 ± 0.26^e^	7.13 ± 1.33^d^	52.80 ± 5.57^a^	19.93 ± 4.54^b^

**Notes:**

Values are means ± S.D. (standard deviation) (*n* = 3–5).

Different lowercase letters indicate significant differences in water fraction among different organs of each species at same age. Significance level is at *P* = 0.05.

The dry mass distribution among different organs was similar to that for water storage, except for the high biomass proportion of young age with the stem bark of Chinese fir. Statistically, the dry mass proportion differed among different components (*P* < 0.001 for Chinese fir and <0.001 for Korean larch) (Table 3), which varied with age class (significant biomass component and age class interaction, *P* < 0.05 for Chinese fir and <0.001 for Korean larch). The largest difference among biomass components was in old age Chinese fir and medium age Korean larch.

**Table 3:** Dry mass fraction (%) of Chinese fir and Korean larch at different age classes.

Species	Age class	Leaf	Live branch	Dead branch	Stem bark	Stem xylem	Root system
Chinese fir	Young	20.45 ± 3.82^ab^	9.50 ± 3.30^b^	0.00 ± 0.00^b^	15.49 ± 3.21^ab^	38.39 ± 6.25^a^	16.17 ± 3.96^ab^
	Medium	9.43 ± 3.52^c^	8.36 ± 2.32^bc^	1.32 ± 0.53^c^	9.96 ± 1.30^b^	51.96 ± 5.80^a^	18.98 ± 4.18^ab^
	Old	2.50 ± 0.44^c^	5.45 ± 0.90^bc^	4.10 ± 1.35^c^	11.50 ± 0.60^b^	62.82 ± 4.57^a^	13.63 ± 1.76^b^
Korean larch	Young	15.88 ± 3.68^b^	20.71 ± 3.99^ab^	0.78 ± 0.71^c^	9.58 ± 2.02^b^	34.50 ± 4.18^a^	18.55 ± 1.79^ab^
	Medium	2.75 ± 1.09^c^	8.33 ± 1.52^b^	4.24 ± 2.08^c^	8.31 ± 1.29^b^	60.18 ± 4.73^a^	16.19 ± 3.57^b^
	Old	4.34 ± 1.17^d^	11.96 ± 2.10^bc^	1.66 ± 0.76^e^	6.66 ± 0.80^cd^	57.88 ± 5.42^a^	17.49 ± 3.05^b^

**Notes:**

The standard error is S.D. (*n* = 3–5).

Different lowercase letters indicate significant differences in dry mass fraction among different organs of each species at same age. Significance level is at *P* = 0.05.

### Water content ratio

**Figure 2:**
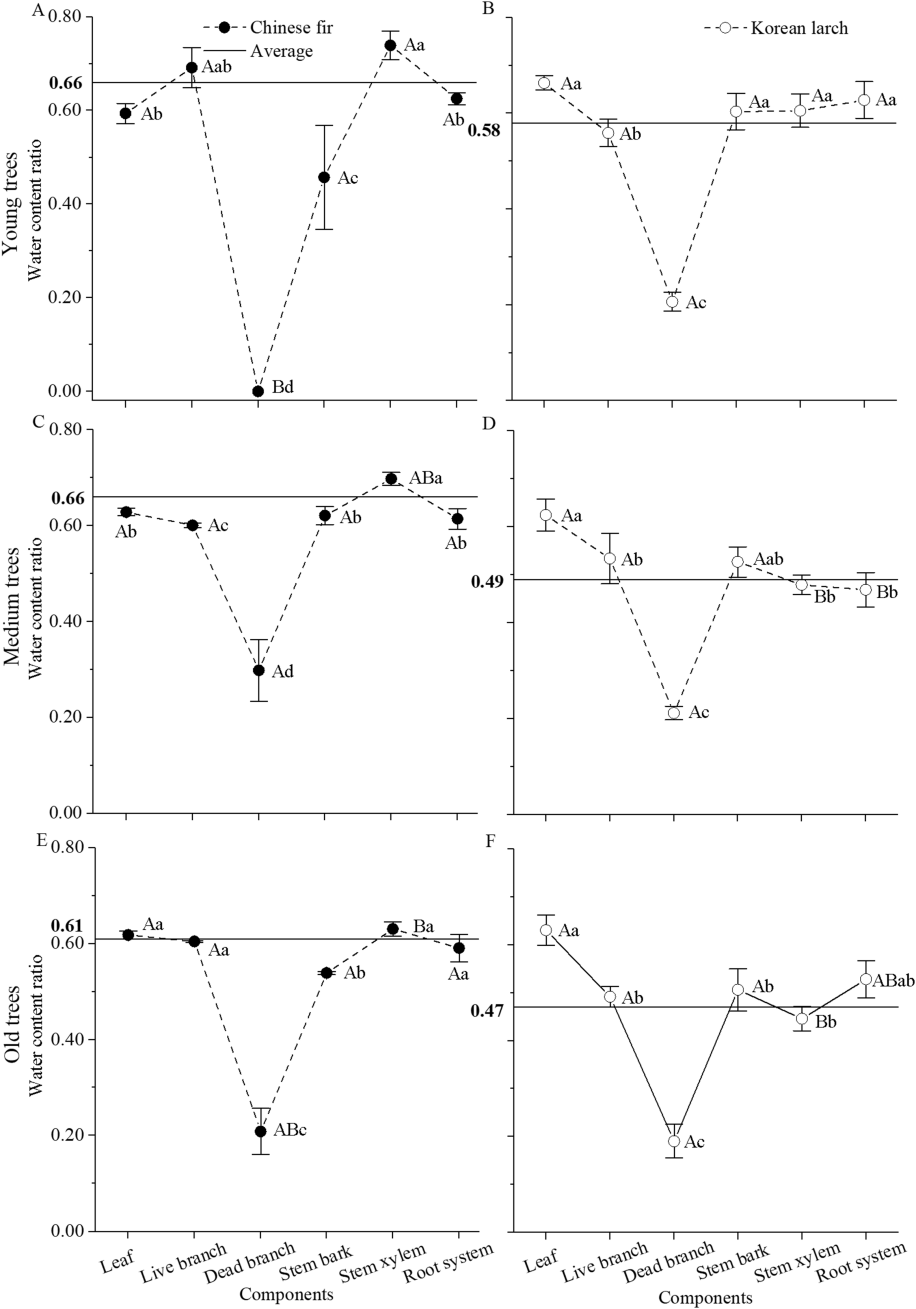
Water content ratio in different structural components of Chinese fir and Korean larch trees by different age classes (±S.D.). The horizontal straight line represents the average water content ratio for Chinese fir (A, C, and E) and Korean larch (B, D, and F). Different lowercase letters indicate significant differences in water content ratio among different organs of each species at same age. Different uppercase letters indicate significant differences in water content ratio of same organ among different age classes of each species. Significance level is at *P* = 0.05.

In both species, tissue water content ratio significantly differed among biomass components (*P* < 0.001 for both Chinese fir and Korean larch) and age classes (*P* < 0.05 for Chinese fir and *P* < 0.001 for Korean larch), and the differences among biomass components in Chinese fir also varied with age class, especially for dead branch and stem xylem (*P* < 0.001 for interaction) (Fig. 2). In general, water content ratio was higher in Chinese fir than in Korean larch and decreased with age in both species. However, water content ratios were similar among different organs of Chinese fir trees at different ages and lower in woody organs in Korean larch trees at older ages, with the exception of necromass. Among different organs, water content ratio was generally highest in stem xylem with Chinese fir and in leaves with Korean larch, and lowest with dead branches in both species.

### Relationships between water storage and dry mass

**Figure 3:**
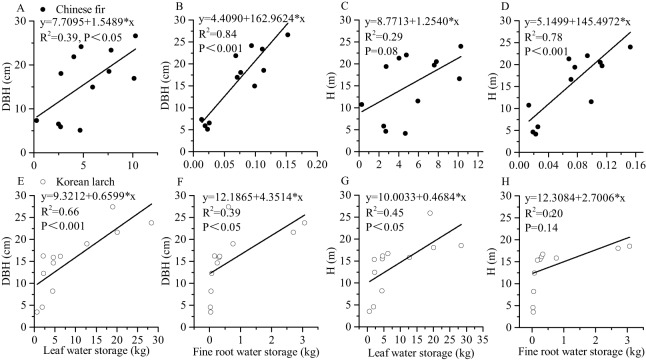
Relationships of leaf and fine root water storage with DBH and H of Chinese fir and Korean larch trees. Significant relationships between the structural component (leaf or fine root) water storage and tree attribute (DBH or H) of Chinese fir (A–D) and Korean larch (E–H) are presented with a solid line.

**Figure 4:**
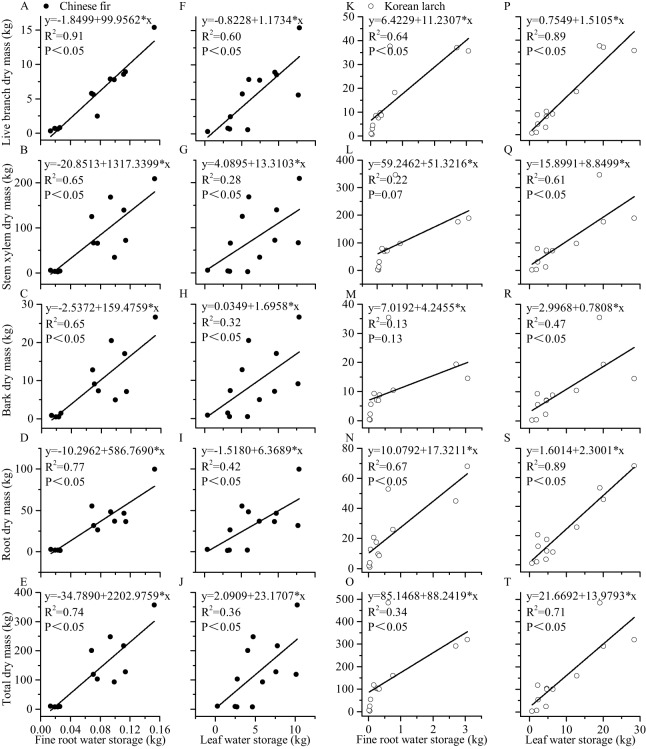
Relationships between leaf and fine root water storage and structural components dry mass of Chinese fir and Korean larch trees. Significant relationships between the structural component (leaf or fine root) water storage and structural component (live branch, stem xylem, stem bark, root, or total) dry mass of Chinese fir (A–J) and Korean larch (K–T) are presented with a solid line.

Both leaf and root water storage increased with DBH and H significantly (Fig. 3). Using the same DBH (or H), Chinese fir showed lower fine root (or leaf) water storage than Korean larch, although the differences among slopes were not statistically significant in either species. Similarly, using the same DBH (or H), fine root showed relatively lower water storage than in leaves for the two conifers. There were differences in the positive linear correlation between fine root water storage, leaf water storage, and component dry mass for the two conifers (Fig. 4). With the same component dry mass, Chinese fir showed lower fine root (or leaf) water storage than Korean larch based on the differences in the linear regression coefficient. Similarly, with the same component dry mass, fine root showed relatively lower water storage than leaves for the two conifers.

**Figure 5:**
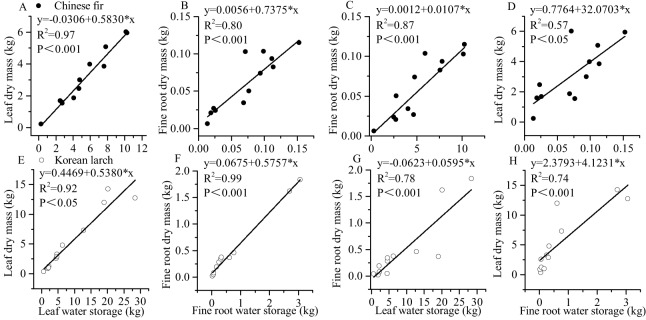
Relationships between water storage and dry mass in leaves and fine roots of Chinese fir and Korean larch. Significant relationships between the structural component (leaf or fine root) water storage and structural component (leaf or fine root) dry mass of Chinese fir (A–D) and Korean larch (E–H) are presented with a solid line.

There were significant positive linear regressions between leaf dry mass and leaf water storage and between fine root dry mass and fine root water storage in both conifers (Fig. 5). The linear correlation between fine root dry mass and fine root water storage of Korean larch was the strongest (*P* < 0.001, *R*^2^ = 0.99), and the linear correlation between leaf dry mass and leaf water storage of Chinese fir was also strong (*P* < 0.001, *R*^2^ = 0.97). Interestingly for both conifers, there was also a strong linear correlation between fine root dry mass and leaf water storage (*P* < 0.001, *R*^2^ = 0.87 and *P* < 0.001, *R*^2^ = 0.78, respectively). The slope of the relationship between leaf dry mass and fine root water storage of Chinese fir was relatively higher than that of Korean larch. In contrast, the slope of the relationship between fine root dry mass and leaf water storage in Chinese fir was relatively lower than that of Korean larch. The slopes of the leaf dry mass and leaf water storage and fine root dry mass and fine root water storage (ratio of dry mass and water storage) of both conifers ranged from 0.54 to 0.74.

### Relationships between water content ratio and dry mass

**Figure 6:**
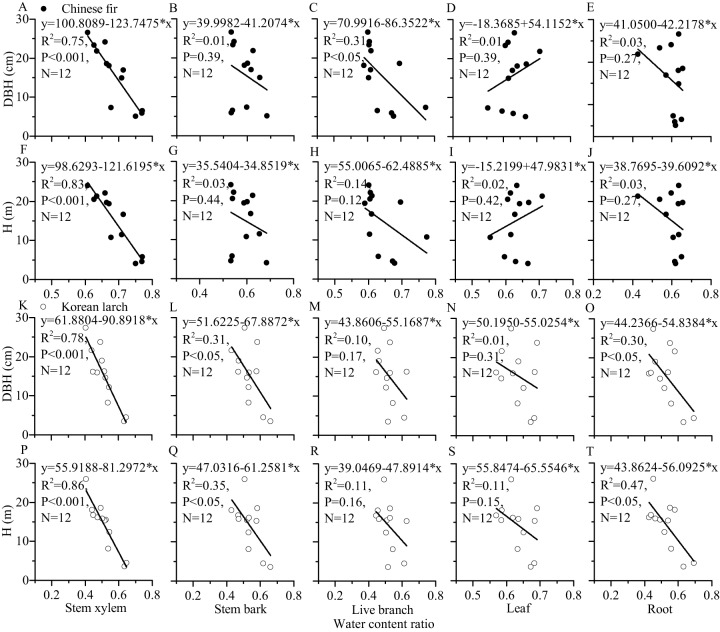
Relationships of structural component (stem xylem, stem bark, live branch, leaf, or root) water content ratio with DBH (or H) of Chinese fir and Korean larch. Significant relationships between the structural component (stem xylem, stem bark, live branch, leaf, or root) water content ratio and tree attribute (DBH or H) of Chinese fir (A–J) and Korean larch (K–T) are presented with a solid line.

**Figure 7:**
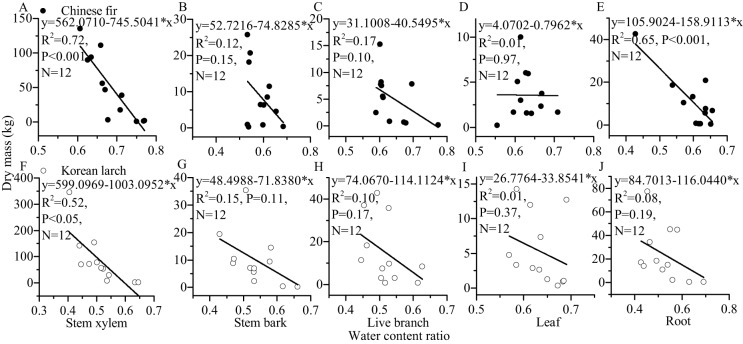
Relationships between structural component (stem xylem, stem bark, live branch, leaf, or root) water content ratio and their dry mass in Chinese fir and Korean larch. Significant relationships between the structural component (stem xylem, stem bark, live branch, leaf, or root) water content ratio and their dry mass of Chinese fir (A–E) and Korean larch (F–J) are presented with a solid line.

For most of the components, the tree sizes (DBH, H, and dry mass components) showed a decreasing trend with the increase of water content ratio component for the two conifers (Figs. 6 and 7). The linear correlation between tree size and stem xylem water content ratio was consistently the strongest for the two conifers. The slopes of the relationship between tree size and stem xylem water content ratio were consistently steeper than any other components of the two conifers. The slopes of the relationship between DBH (or H) and stem xylem water content ratio of Chinese fir were relatively higher than those of Korean larch. In contrast, the slopes of the relationship between stem xylem dry mass and stem water content ratio were higher than those of Chinese fir. In general, there was no significant linear correlation between tree size and water content ratio for many components of the two conifers.

## Discussion

### Distributions of water storage and dry mass

Water storage is an important physiological and biochemical index in plants. Our results demonstrated that the water distribution in the two conifers were similar (Table 2), indicating that there are similar rules for conifer species with no water stress, regardless of climate and tree species. The experimental results also showed that the water storage of Chinese fir and Korean larch was mainly distributed in the stem xylem. This was consistent with the results of Tian et al.’s (2018) results in temperate forests of African woodland regions and tropical woodlands in the south-eastern USA. The dry mass of the two conifers was also mainly distributed in the stem xylem. The water storages of the leaf and root systems were proportional to their dry mass proportions. Regression analyses showed that the dry mass and water storage of the leaves and fine roots of the two conifers were significantly proportional, and the slopes (ratio of dry mass and water storage) were close to one. The results were consistent with the relative relationship between the proportions of water storage and dry mass, indicating the reliability of the experimental results. The results of the stem xylem dry mass were directly proportional to the stem xylem water storage (Tables 2 and 3). This was consistent with the results of Becker et al.’s (2012) research on 23 angiosperm tree species (*Alstonia boonei* et al.) in East African rain forests, which found a significant proportional relationship between the specific gravity of wood and water storage. This may be partly due to the importance of water to plant physiological and biochemical reactions (Guada et al., 2018). In total, the two typical coniferous species had similar water storage distribution characteristics, which supported biomass accumulation (a proxy for productivity) at the individual tree level.

### Effects of age class and component on water storage

The statistical results of our study showed that both component and age class had an effect on the water storage of the two conifers. At the young tree stage, the water storage proportions of vegetative organs (leaf and root system) were relatively high (Table 2). This might be due to the nutritional requirements (CO_2_ and inorganic fertilizers) of the growth of the two conifers. A relatively high proportion of water storage was allocated to vegetative organs, which may be more conducive to nutrient uptake by young trees (Lisar et al., 2012). With increasing age, the water storage proportion in stem xylem increased relative to that in the other organs, except in Korean larch in the old tree stage (Table 2). This might be due to the demand for mechanical support from tree crowns (Hoeber et al., 2014), which might promote the development of stem xylem. At the same time, the stem xylem played an important role in storing and transporting plant water (Von Allmen, Sperry & Bush, 2015; Saito et al., 2016).

Additionally, there were some differences, such as stem xylem and root system, in component water storage between the two conifers (Table 2). Water content ratios were similar among different structure components of Chinese fir trees at different ages and lower in stem xylem in Korean larch trees at older ages, with exception of necromass (Fig. 2). These differences may indicate the differences in biomass productivity between the two species (Oliva Carrasco et al., 2014). Specifically, the water status difference in stem xylem of the two species may indicate that Chinese fir has a relative higher wood production function than Korean larch. These differences may be due to the contrasting climates, in which precipitation and temperature during the growing period could significantly affect the growth, and hence the water requirements, of the trees. The meteorological data on the sampling sites and periods showed that both the temperature and precipitation were normal. The temperature and precipitation of JF were both higher than those of DF. Paz-Kagan & Asner (2017) reported that the canopy water storage change in Mediterranean-type communities was much more closely related to environmental factors than to the species composition in the northern portion of California, USA. The average temperature and precipitation might partly contribute to the water storage difference between the two conifers. Moreover, the two conifers belong to species with different successional status. Species with different successional statuses might have inherently different water storage distributions, which might partly lead to the observed difference. It would be best to compare the same species, or species with the same successional status, living in contrasting climates, but there is no such a species. If trees of the same types are chosen, the difference in component water storage may be lower than those of different species. Although different climatic conditions and successional statuses affect both conifers, the consistently higher stem xylem water storages in both conifers illustrated their similar water use and storage strategies. Additionally, considering that the selected two conifers were typical, local, fast-growing coniferous species in two different climate regions, the results of our study might help to better understand the relationship between water storage and dry mass component for conifers, regardless of the climatic conditions and species.

### Regression analyses of biomass accumulation and fine root

Fine roots play an important role in forest carbon flux and nutrient and water acquisition. Leaves are also important physiological organs in plants. However, information on the relationship between fine root and leaf water storage remains scarce. Regression analyses have determined the interspecific relationships between leaf and fine root traits in order to better understand plant strategies of resource acquisition (Murty, McMurtrie & Ryan, 1996; Magnani, Mencuccini & Grace, 2000; Li & Bao, 2016). Our results indicated the similar water related characteristics and their close relations to biomass accumulation and growth in both fast growing species at contrasting climates first, illustrating the same coherent strategies of fast growing conifers in water utilization. Such result was demonstrated by significant relationships between fine root water and component biomass, growth and especially leaf mass and water traits, as fine root is the important organ for water uptake and foliage is the important organ for carbon sequestration.

Our study found that there was a strong positive linear correlation between the dry mass of the fine root and leaf of Chinese fir and Korean larch. A similar relationship has been reported for some other forest species (*L. gmelinii*, *Spiraea ussuriensis*, *Sorbaria sorbifolia* et al.) (Meng et al., 2018; Zhou et al., 2018a). In a previous study, Santantonio reported a linear relationship between fine root biomass and leaf biomass in several conifer species (*Abies amabilis*, *Picea sitchensis*, *Pinus contorta*, etc.) (Dan, 1989). Strong linear relationships between leaf biomass and fine root biomass were also found for *Eucalyptus globulus* (O’Grady, Worledge & Battaglia, 2006) and *Pinus tabuliformis* (Jia, Liu & Li, 2015). Zhou et al. (2018a) also found that there was a significant linear relationship between tree fine root biomass and leaf biomass for coniferous species. These results strongly support the hypothesis that fine roots were related to leaf biomass and functional equilibrium relationships (Brouwer, 1963; Mäkelä & Sievänen, 1987).

Furthermore, our study also found a significant linear relationship between fine root dry mass and leaf water storage for both Chinese fir and Korean larch. This is partly because the root system is the main organ for plant water absorption, the leaf is the main organ of photosynthesis, and the fine roots provide water for photosynthesizing leaves (Zhou et al., 2018a). Therefore, the fine root biomass and leaf water storage maintain a dynamic balance. The relationship between leaf water storage and fine root dry mass is not often calculated, making comparisons with the results of other studies difficult. Our results may also support the functional equilibrium relationships between fine roots and leaves.

Diameter at breast height and H are proxies for biomass. Relationships between tree fine roots and tree attributes are important for the establishment of tree-level fine root biomass models (Zhou et al., 2018a). The results highlighted that the linear relationships between fine root water storage and DBH and H were stronger for Chinese fir, while the linear relationships between leaf water storage and DBH and H were stronger for Korean larch. This indicates that the fine root water storage of Chinese fir is closely related to biomass, while the leaf water storage in Korean larch is closely related to biomass. The results were consistent with Fig. 4. Additionally, according to the scatter plot (Figs. 3E–3H), other types of relationships may be more appropriate for Korean larch. We can deduct similar trend of biomass and water content relationships as those of growth-water for Chinese fir and Korean larch, respectively, evidenced by different *R*^2^*s* (Figs. 3–5), indicating the importance of fine root water to Chinese fir and leaf water to Korean larch, respectively.

## Conclusions

The results of this study indicate the similar water related characteristics and their close relations to biomass accumulation and growth in both fast growing species at contrasting climates, illustrating the same coherent strategies of fast growing conifers in water utilization. The fine root is the main water absorption component for the two conifers and determined the leaf mass and water traits. The fine root and leaf biomass coordinated with biomass productivity. For the two conifers, water storage was basically proportional to biomass component with no water stress, regardless of different climatic conditions and tree species. The higher water storage in its stem xylem may support Chinese fir to have a relatively higher wood production function than Korean larch does.

In future research, the dynamic characteristics of the relationship between water storage and biomass components caused by environmental factors in diverse forest types should be further investigated.

## Supplemental Information

10.7717/peerj.7901/supp-1 Supplemental Information 1 The raw data of water storage and biomass components for Chinese fir and Korean larch. The fresh weight, dry mass, water content ratio, and water storage components for Chinese fir and Korean larch are shown.

## Additional Information and Declarations

### Data Availability

The following information was supplied regarding data availability: Raw data is available as Dataset S1.
